# Cause-specific mortality transition among women of reproductive age with special reference to maternal mortality: the Magu health and demographic surveillance system, Tanzania, 1995–2022

**DOI:** 10.1080/16549716.2025.2611693

**Published:** 2026-03-18

**Authors:** Milly Marston, Sophia Kagoye, Jacqueline Materu, Charles Mangya, Jim Todd, Mark Urassa, Ties Boerma

**Affiliations:** a Department of Population Health, London School of Hygiene & Tropical Medicine, London, UK; b National Institute for Medical Research Mwanza Research Centre, Mwanza, Tanzania; c Department of Epidemiology and Biostatistics, Catholic University of Health and Allied Sciences, Mwanza, Tanzania; d Institute for Global Public Health, University of Manitoba, Winnipeg, Canada

**Keywords:** Tanzania, mortality, maternal mortality, cause of death, population cohort

## Abstract

**Background:**

Limited population data exist on mortality and causes of death among women of reproductive age, including maternal mortality.

**Objective:**

To present trends from 1995 to 2022 in mortality, cause of death, maternal mortality ratio and place of death from the rural Magu health and demographic surveillance site in north-west Tanzania.

**Methods:**

Data on residency, fertility, and verbal autopsy were analysed to compute trends in all-cause and cause-specific mortality for women 15–49 years, using InSilicoVA, a Bayesian probabilistic model. Maternal mortality was estimated for three periods: 1995–2002, 2005–2011 and 2015–2022. We described place of death and healthcare utilization leading up to death by calendar time and broad cause of death.

**Results:**

All-cause mortality among women 15–49 declined from a peak of 9.0 deaths per 1000 person-years in the late 1990s to 3.5 in 2020. HIV/TB contributed 61% of this reduction. During 1995–2022, infectious diseases were the leading cause (54%), followed by NCD (29%). Maternal mortality declined but stagnated from approximately 2010 at 281 per 100,000 live births in 2015–2022 and remained a leading cause of death (12%). Comparing 2009–2015 and 2016–2022, more deaths occurred in health facilities (37.3%–45.9%), and more women sought care during terminal illness or maternity (85%–93%).

**Conclusion:**

Despite a major reduction in all-cause mortality among women 15–49 years, mainly due to decreased HIV/AIDS deaths, infectious diseases remain the leading cause alongside a much-increased share of NCD. While maternal mortality levels have decreased, its share of total deaths has remained unchanged compared to 25 years ago.

## Paper Context

- **Main findings:** All-cause mortality among women of reproductive ages declined almost threefold in the Magu Health and Demographic Surveillance Site (HDSS) in northwest Tanzania during 1995–2022, mainly driven by a decline in HIV mortality during 2000–2014 while maternal mortality remained a major cause of death
- **Added knowledge:** The study contributes to the major gap in population data on long-term mortality trends by cause among women in sub-Saharan Africa
- **Global health impact for policy and action:** HDSS are a critical source of population-based cause of death data, informing health strategies, in countries with poorly functioning vital statistics systems.

## Background

Global estimates and several country studies have shown a major mortality transition for adult women over the past three decades. In sub-Saharan Africa, the highest mortality region in the world, mortality among women 15–49 years is estimated to have declined by almost 40% between 1990 and 2020 [1]. In addition, cause-specific mortality patterns among women of reproductive age have also shifted. According to WHO estimates for the African region, the share of infectious diseases among female deaths 15–49 years between 2000 and 2020 declined from 61% to 40%, while the contributions of non-communicable diseases and injuries increased from 14% to 32% in 2020 to 6% to 9% in 2000, respectively [2]. The share of maternal conditions, however, did not decline and remained at 19% of deaths among women 15–49 years, even though total fertility declined from 5.7 to 4.5 children per woman during 2000–2020 period. This persistence underscores maternal mortality as a continuing priority in the region, especially in light of Sustainable Development Goal 3.1, which calls for reducing the global maternal mortality ratio to fewer than 70 per 100,000 live births by 2030 [3].

Estimates for Tanzania show an even more pronounced transition. Mortality among women 15–49 years in Tanzania was 52% lower in 2020 than in 1990 and more than 60% lower than in 2000 when HIV mortality was high. The share of infectious diseases decreased from 72% in 2000 to 48% in 2020, but little change was observed in the share of maternal conditions (16% in 2020) [2]. However, the estimated maternal mortality ratio (MMR) has declined significantly from 760 (80% uncertainty interval: 615,933) to 238 (174,381) per 100,000 live births, according to UN estimates [3]. Total fertility also declined from 5.7 to 4.8 children per woman [1].

The estimates for Tanzania, as in most other countries in the region, are underpinned by data from household surveys with sibling survival histories, notably the demographic and health surveys (DHS) [4], and, to a lesser extent, censuses. Since 1996, five national surveys have provided data on adult mortality levels and maternal mortality. The empirical data on a cause-of-death transition are much more limited, since death registration systems with reliable cause of death are poorly functioning in most countries in sub-Saharan Africa, including Tanzania [5].

Longitudinal community studies, generally referred to as Health and Demographic Surveillance Sites (HDSS), have generated empirical data on population adult mortality, as well as causes of death through verbal autopsy [6–13]. In general, the results support a major decline in adult female mortality driven by a decline in infectious diseases over the last two to three decades [8–13]. However, marked differences in this epidemiological transition have been observed, partly due to differences in mortality levels and fertility, epidemiological conditions (e.g. malaria and HIV), and socioeconomic characteristics.

HDSS may also provide data on maternal mortality data. A synthesis of data from 12 HDSS in eight countries in sub-Saharan Africa showed that the MMR varied from 128 to 461 per 100,000 live births (median: 222) and the maternal mortality rate was 0.15–0.74 per 1000 person-years (median: 0.30) [14]. However, this should be considered an underestimation. In addition to common challenges in identifying early maternal deaths and indirect maternal deaths, HDSS data completeness tends to be variable due to difficulties in capturing pregnancy status [15–17]. A recent study of nine HDSS in eastern and southern Africa showed that maternal mortality rates had declined in four sites but remained similar in the others [10].

The Magu Health and Demographic Surveillance Site (HDSS) is unique as the longest-running community study in Tanzania with data on mortality by cause, including maternal mortality over almost three decades. In this study, we assessed the mortality transition among women 15–49 years during 1995–2022, with a focus on maternal mortality.

## Methods

### Data

The Magu HDSS is situated in rural north-west Tanzania. It was established in 1994, primarily as an observational HIV population cohort study. The baseline population was 19,347, growing at an average of 3.8% per year to 54,024 in 2022 [18]. This study is an open community cohort covering all households in nine contiguous villages of one administrative ward (Kisesa) in the Magu district. The HDSS area is predominately rural, with a semi-urban trading center located on the main road between Mwanza City and the Kenyan border. Regular demographic surveillance collected basic data on births, deaths, and migration, along with other events, at an average interval of 8 months. Details of the HDSS design are described in detail elsewhere [18,19]. Previous research has shown that the study population is typical of mainland Tanzania in terms of socioeconomic, demographic, and health indicators [18]. HIV prevalence among women 15–49 years has increased from 6.6% in 2004 to 7.2% in 2016 [18]. Written consent was obtained from participants.

When a death was recorded in the HDSS demographic rounds, A clinical officer typically visited the household approximately 4 months after the death to conduct a verbal autopsy (VA). The VA included both a structured questionnaire and a narrative section to document the events leading up to death. Since the establishment of the HDSS, there have been three types of VA questionnaires: a project-specific questionnaire between 1995 and 2002, followed by the INDEPTH (The International Network for the Demographic Evaluation of Populations and their Health) standard questionnaire between 2003 and 2007 [20] and the WHO standard questionnaire from 2008 [21,22]. For most of 2003 and 2004, VA was not conducted due to project transitions. The questionnaires were standardized into a data specification provided by the ALPHA Network (ALPHA: Analysing Longitudinal Population-based HIV/AIDS Data on Africa) [23,24]. The InsilicoVA inputs are shown in Supplementary materials p3 showing the difference between pre and post WHO questionnaire.

## Analysis

### Mortality rates

Mortality rates were calculated for women aged 15–49 years in the HDSS as the number of deaths divided by the number of person-years lived in the HDSS. We excluded deaths and person-years of migrant individuals while they were outside the study area. Age-specific mortality rates were calculated by five-year age groups. Annual mortality rates were calculated, and trends are analyzed using a smoothed 5-year running average to minimize the impact of heaping of deaths due to the timing of survey rounds and random fluctuations. The HDSS is not a sample, as everyone within the area is included in the analysis. Therefore, we do not present confidence intervals.

### Cause of death

Linking the VA and residency datasets, we calculated VA coverage in the HDSS area over time. The cause of death was ascertained using the openVA package in R (4.0.3) using the InSilicoVA algorithms from the openVA package (1.0.14) [25]. InSilicoVA identifies the joint distribution of individual causes of death and population-level cause-specific mortality fractions (CSMFs) from verbal autopsy data, generating probabilistic estimates for both individual causes and population-level CSMFs. Specific causes of death from InSilicoVA were categorized into four broad groups: communicable, HIV/TB, non-communicable, maternal causes and external causes [10] (supplementary materials p4). The probabilities for each cause of death were aggregated to give a population level distribution of the cause of death by four-year periods between 1995 and 2022 and pregnancy status. We applied this distribution to the all-cause mortality rates among women 15–49 years in the HDSS area to provide cause-specific mortality rates for each four-year period. We assumed that the distribution of cause of death for those without VA was the same as those in the same broad age group and period of the death.

### Maternal mortality and related variables

Pregnancy-related death is defined as the death of a woman during pregnancy or within 42 days of termination of pregnancy, irrespective of the duration and site of the pregnancy, from any cause. Maternal death is defined as the death of a woman during pregnancy or within 42 days of termination of pregnancy, irrespective of the duration and site of the pregnancy, from any cause related to or aggravated by the pregnancy or its management, but not from unintentional or incidental causes [26]. To distinguish maternal deaths from pregnancy-related deaths, we used the most probable broad cause of death assigned to each woman by InSilicoVA, and any that were categorised as external were excluded.

To ascertain pregnancy status at death, we used the VA data, which included a question on pregnancy status at the time of death and, if so, how many months or if they died within 6 weeks of giving birth. We calculated the proportion of pregnancy-related deaths by dividing the number of deceased women who were pregnant or postpartum by all deceased women within a given time period for all women 15–49 with a VA.

The pregnancy-related mortality rate was defined as the number of deaths to pregnant/postpartum women aged 15–49 divided by the number of person-years in a given period. Since VA coverage was incomplete, we assumed that the proportion of maternal deaths by 5-year age group and place of residence in a given period was the same as those who had known pregnancy status.

The pregnancy-related mortality ratio (PRMR) is defined as the number of pregnancy-related deaths during a given time period per 100,000 live births during the same time period. To obtain the number of live births, we computed the fertility rate of women aged 15–44 and 15–49, defined as the number of live births in the period born to women within the age group, divided by the number of person years person-years lived in the HDSS by women of the same age group, in the same period, using survival analysis. We assessed the completeness of birth reporting by comparing our results with DHS, which were available for 15–44-year-olds. Where there was evidence of underreporting of births, we imputed the fertility rate by fitting a straight line to the smoothed annual fertility rates. The maternal mortality ratio (MMR) is defined as the PRMR but includes only maternal deaths in the numerator, as defined above.

We conducted a sensitivity analysis to assess how mortality changes with different assumptions regarding the cause of death and pregnancy status. First, we calculated the MMR if we assumed that all women who died with unknown pregnancy status were not pregnant. Second, we estimated the MMR using a 20% change in the estimated number of live births in each period. These estimates were used as error bounds for the estimated MMRs for Magu HDSS.

We selected three periods, 1995–2002, 2005–2011 and 2015–2022, to calculate the MMR and related variables in order to ensure adequate numbers and high VA coverage. This omits the period 2003–2004 where there was no or very low VA coverage.

All estimates of all-cause and maternal mortality were compared with the results of the national Demographic and Health Survey in Tanzania [27].

### Place of death and care sought prior to death

Data on place of death and care seeking were available only from 2007. Using VA, we assessed the trends in place of death, by pregnancy status, calendar period, and broad cause of death from 2009 to 2022. We examined whether the deceased sought care at formal or informal health services during the terminal illness.

### Verbal autopsy narratives

We looked further into pregnancy-related deaths using the verbal autopsy narratives between 2015 and 2022 when the data were available to further examine the data quality and to highlight the circumstances around each pregnancy-related death. Two medical doctors reviewed the signs and symptoms recorded in the questionnaire and the narrative to assign a specific cause of death.

## Results

### Mortality and cause of death

**Figure 1.**
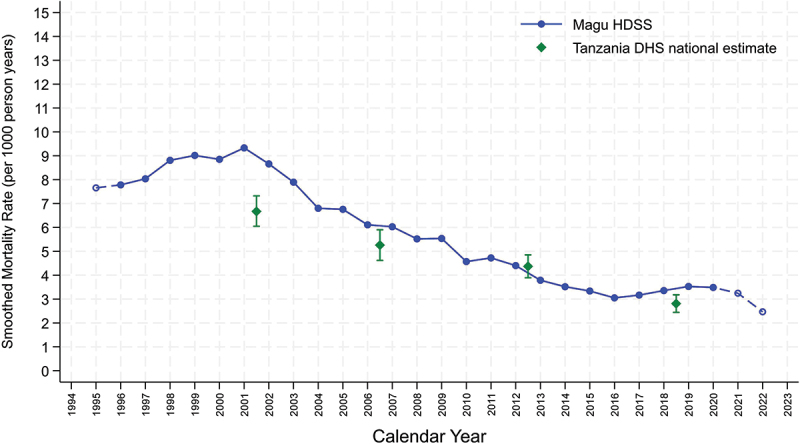
Age adjusted mortality rates for women 15–49, Magu HDSS, by calendar time using a five year moving average, 1995–2022, (dashed lines denote one year point estimate) compared to national estimates from the Tanzanian DHS.

From 1995 to 2022, there were 940 deaths, and 177,729 person years lived in the study area for women aged 15–49. The mortality rate in women aged 15–49 reached a plateau of almost 9 per 1000 person-years during 1998–2002 followed by a steady decline of 60% until 2014, when the decline stagnated at around 3.5/1,000 person-years until 2020 (Figure 1, supplementary materials p5).

VA coverage for women aged 15–49 varies over calendar time above 80% in the late 90s dropping to around 60% in the early 2000s. 2003 and 2004 saw very low coverage since then coverage has ranged from 50% to 100% up to 2022 (Supplementary materials p6-7). Pregnancy status was unknown in some VA yielding a slightly lower proportion of women with known pregnancy status compared to VA coverage. The percentage of women who had a known pregnancy status at the time of death (excluding 2003 and 2004) were 69.5%, 74.8%, and 84.0% in 1995–2002, 2005–2011 and 2015–2022, respectively.

**Figure 2.**
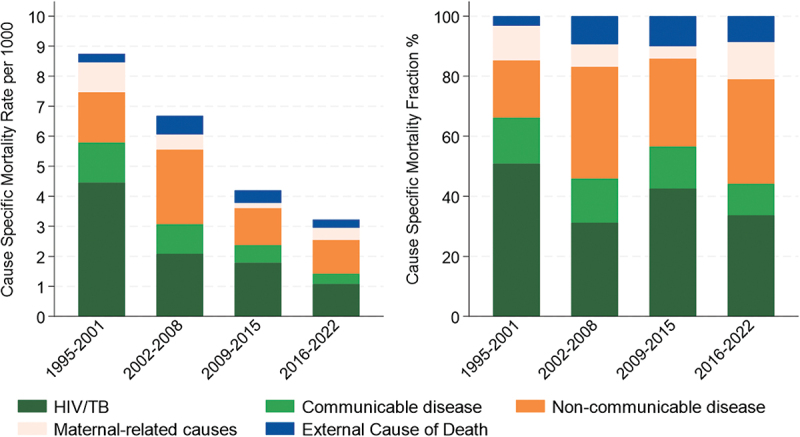
Cause-specific mortality fraction and cause-specific mortality rates by calendar period for women aged 15–49 years, Magu HDSS, 1995–202.

The overall mortality decline was driven by a large decline in the HIV/TB mortality rate reducing from 4.46 per 1000 in 1995–2001 to 1.09 per 1000 in 2016–2022 and to a much lesser extent, other causes (Figure 2, supplementary materials p8). This corresponds to 61% of the all-cause mortality decline, followed by 18% from other communicable diseases, 11% from maternal causes, 10% from NCD, and none from external causes. The contribution of maternal-related cause of death to all deaths among women 15–49 years was similar in the first and last periods at 11.5% and 12.4%, respectively.

These changes have resulted in much greater prominence of NCDs and external causes of death, accounting for 34.8% and 8.6% of all deaths in the most recent period 2016–2022 compared to 19.0% and 3.2% in 1995–2001, respectively. For NCDs in the most recent period, the largest cause-specific mortality fractions came from neoplasms (Supplementary Material p9).

Despite the shift in cause-specific mortality fractions, primarily due to the major decline in infectious diseases, HIV/TB (33.7%) and other communicable diseases (10.5%) remained the leading cause group over all periods (declining from 66.3% to 44.2% of all deaths from 1995–2001 to 2016–2022).

### Maternal mortality measures

To match data availability, we used three periods for the analysis of pregnancy-related mortality. There was only one pregnancy-related death that was not maternal in the 2005–2011 period, caused by rabies from a dog bite. Therefore, pregnancy-related and maternal measures are equivalent for the periods 1995–2002 and 2015–2022.

**Figure 3.**
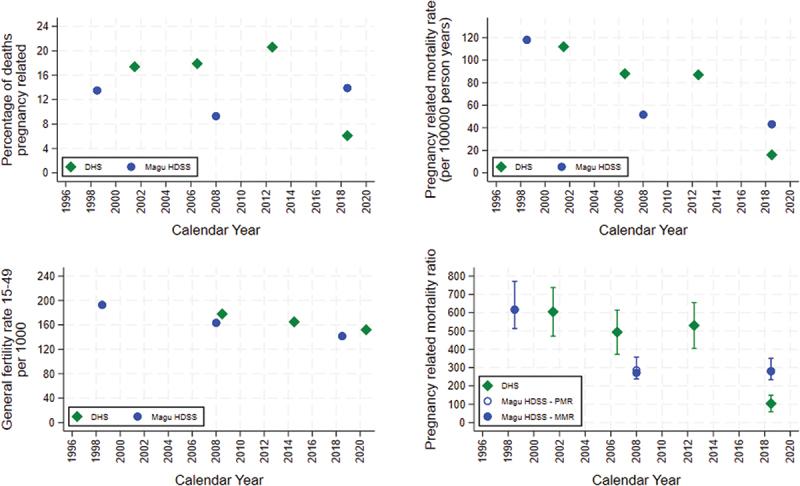
Pregnancy mortality related measures plotted at the midpoint of the period of estimation a) top left-proportion of women aged 15–49, who were pregnant or postpartum at time of death, for those with a known pregnancy status. b) top right-estimated pregnancy related mortality ratio using imputed pregnancy status for those of unknown pregnancy status at death, c) bottom left-fertility rate women aged 15–49 using imputed fertility rate for the last period for Magu HDSS. d) bottom right -the maternal and pregnancy related mortality ratio using the imputed pregnancy status for those with unknown pregnancy status at death and the imputed births derived from the fertility rates in (c), note that the MMR for DHS Tanzania is the same as the PMR for the latest period 2015–2022. Also the MMR and PMR are the same for the Magu HDSS for the earlier and later time point. With 95% confidence intervals for DHS and error bounds for Magu HDSS based on sensitivity analysis around unknown pregnancy status, see supplementary materials P12.

The proportion of deaths among women 15–49 that were pregnancy-related ranged from 9.3% to 13.9% over the three time periods from 1995 to 2022 (Figure 3(a)). The age groups with the highest percentage of pregnancy-related deaths were in the 20–29 age range for the 2005–2022 period, in the earlier period, the highest pregnancy-related deaths were more dispersed in the 15–19, 20–24 and 35–39 age group (Supplementary materials p10).

Assuming that the pregnancy status distribution is the same in the unknown group by five-year age group and place of residence in the given periods, pregnancy-related mortality was 118, 52, and 43 per 100,000 person years in–1995–2002, 2005–2011 and 2015–2022, respectively (Figure 3(b)).

The general fertility rate (GFR) for women aged 15–44 in the Magu HDSS is similar to the national Tanzanian estimate from the DHS at around 200 births per 1000 person-years in the late 1990s and the early 2000s, reducing to approximately 160 per 1000 in 2020. However, between 2011 and 2015, and in 2022, there was an under enumeration of births in the HDSS (Supplementary materials p11). Imputed estimates of the GFR for women 15–49 years, required for maternal mortality calculations, were performed for these years and are shown in Supplementary Material p11. The resultant GFR of 15–49 in the three periods is very similar to the national survey, although slightly lower in the 2015–2022 period at 142 per 1000 person years compared to 152 in the DHS (Figure 3(c)).

The MMR estimate was highest in 1995–2002 at 617, declining to 272 in 2005–2011 and 281 in 2015–2022 (Figure 3(d)). The pregnancy-related mortality ratio in Magu HDSS 1995–2002 was in the same ballpark as the national DHS results for a similar period (617 and 605, respectively). However, the pregnancy-related mortality ratio then declined quicker than the DHS and was lower at 281 in 2005–2011 compared to 494 in the DHS in 2003–2010. The latest directly comparable period 2015–2022, where for both the DHS and Magu HDSS, the pregnancy-related mortality ratio is the same as the MMR, the Magu HDSS MMR is much higher than the results of the DHS 2022 (281 compared to 104).

For the sensitivity analysis, we assume as an upper estimate an overestimate of the fertility rate of 20%. For the lower estimate, we assumed both no extra maternal deaths in those with pregnancy status unknown and an underestimate of fertility of 20%. For the latest period 2015–2022 this yielded a lower estimate of the MMR of 205 and an upper estimate of 351 (supplementary materials p12).

#### Data confirmation using verbal autopsy narratives

To both understand the circumstances around maternal deaths and assess the reliability of the pregnancy status information, we included selected VA variables with the narratives of the most recent period 2015–2022 in supplementary materials p13-16. The majority of deaths were from direct maternal-related causes of postpartum haemorrhage (7), eclampsia(2), anaemia (1), ectopic pregnancy (1), prolonged obstructed labour (1), two cases were of induced abortion. For other causes two deaths were from HIV, two from pulmonary embolism, and three indeterminate. All but one, who died on the way from the hospital, had either delivered in a hospital or health centre or had gone to hospital to be treated.

### Place of death for women

**Figure 4.**
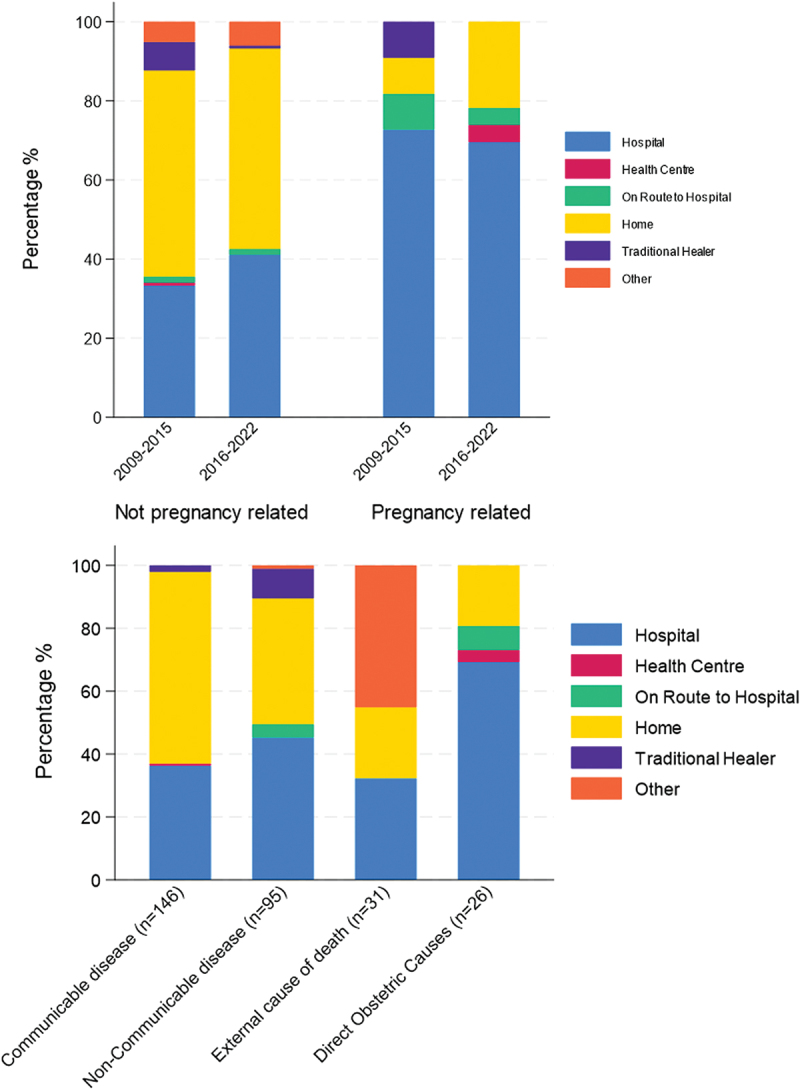
Distribution of place of death for women aged 15-49between 2009–2022, for those who have a VA a) by pregnancy status and period (top). Not pregnancy related *n* = 272, pregnancy related deaths *n* = 34 and b) by cause of death, excluding indeterminate cause of death (bottom).

Overall, the proportion of deaths with a VA that occurred in health facilities increased from 37.3% in 2009–2015 to 45.9% in 2016–22. For women whose death was not pregnancy-related, there was an increase in deaths in hospitals over time from 34.1% in 2009–15 to 41.0% in 2016–2022. Deaths at traditional healers reduced from 7.2% in 2009–15 to 0.7% in 2016–2022 (equating to 1 death). Very few deaths occurred in health centres. The majority of pregnancy-related deaths occurred in hospitals, with little change over time (72.7% in 2009–15 to 69.6% in 2016–22) (Figure 4(a), supplementary materials p17).

Most women who died of maternal-related causes between 2009 and 2022 died in hospital (69%), this was lower for communicable diseases and NCD at 36% and 40%, respectively. Figure 4(b) External causes of death accounted for most of those listed as other. For communicable cause of death, home was most common (60%). Death at a traditional healers accounted for a small proportion of deaths (5% overall) and was most prevalent for those who died of NCD (12%).

**Figure 5.**
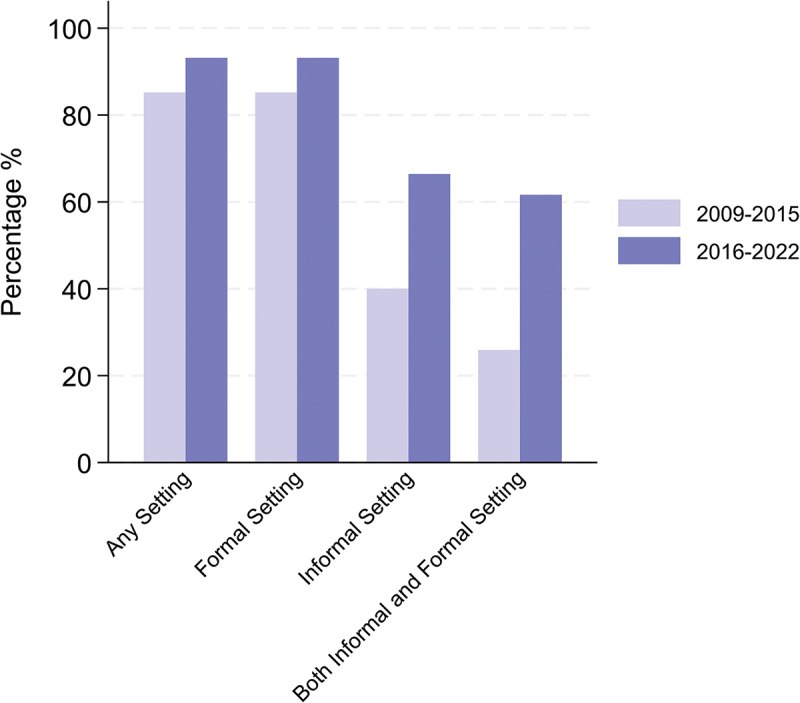
Percentage of women who received treatment for the illness that led up to the death by broad calendar period. This excludes deaths from external causes. (2009–2015 *n* = 135, 2016–2022 *n* = 146).

Accessing treatment from a formal care setting prior to death gradually increased over time from 85.2% in 2009–2015 to 93.1% in 2016–2022 (Figure 5). There was also an increase in treatment in informal settings prior to death, which was mainly due to increased pharmacy use. In 2015–2022, treatment from a formal setting was highest for those who died of a communicable disease at 97.4% and lowest for maternal deaths at 81.0%, presumably due to the short duration of the terminal episode (Supplementary materials p18). There was little difference in treatment in a formal setting leading up to the death for those dying in a formal setting or at home, although those who died at home were more likely to receive informal treatment (85.3% compared to 53.0%, supplementary materials p19).

## Discussion

This study provides a comprehensive look at mortality of women of reproductive age in the Magu HDSS since the nineties. As we have shown elsewhere, Magu HDSS has similar indicators of socioeconomic development, demography, and health and, in many ways, can be considered typical for mainland Tanzania [18]. Five main findings can be excerpted from our analysis:

First, mortality among women 15–49 years declined by 60% since the late nineties. This was driven by a decline in mortality due to infectious diseases, notably HIV mortality (61% of the decline) but also other infectious diseases (18%). In line with epidemiological transition theory and data from other HDSS in sub-Saharan Africa, NCD has become much more prominent as a cause of death. However, there are two caveats. Infectious diseases, led by HIV/TB, still accounted for 44% of all deaths in this age group, which is more than that of NCD (33%). Additionally, the decline in all-cause mortality stagnated from 2014. Both findings stress the need to strengthen a comprehensive primary healthcare approach that addresses all major causes for women of reproductive age [28].

Second, the Magu HDSS data show that, as a cause of death for women of reproductive age, maternal mortality is still as important as 25 years ago in relative terms, despite declines in fertility and risks of maternal death. The only other population-based statistics from longitudinal research studies is from the Ifakara HDSS in east-central Tanzania, this study only provided cause of death together for both sexes and aged 15–59 between 2003 and 2007, but patterns by broad cause were similar [29]. The MMR decline occurred before 2010 but remained at 281 per 100,000 live births in the most recent period. This may be considered surprising given the priority given to maternal health issues in comparison with other health issues for women of reproductive age. Using the distributions of deaths and live births during 2016–2022 (70% of maternal deaths and 84% of live births occurred in health facilities, with births based on a survey in this population [28] and trend in DHS surveys for Mwanza region [30,31]), we estimated the institutional MMR at 234 and the community MMR at 526 per 1000 live births. This points to two obstacles: the quality of care in health facilities, and women delivering at home under high-risk circumstances. The relative importance of maternal causes and the MMR trends indicates the need for continued prioritization of maternal survival.

Third, even though more women of reproductive age died in hospitals than two decades ago, still 46.5% died at home during 2016–2022. This also has implications for strategies to collect data on causes of death. Efforts to improve the use of medical certification of the cause of death in hospitals will continue to be complemented by VA data for community deaths. The Ministry of Health in Tanzania has embarked on large scale VA in two regions. The initial results suggest that this is feasible and may yield valuable data for planning and programming [32].

Fourth, the VA also showed that almost all women had contact with formal healthcare settings during the terminal illness or condition in the most recent period, suggesting that there are ample opportunities to intervene. We did not have information on where failures may lie, whether it is delay in seeking treatment (first phase delay), ability to get to a place of treatment (second phase delay) [33] or inadequate care either due to skills or resources at the health facilities (third phase delay). In a review of hospital records for maternal deaths in 2015/16 in a referral hospital in another region of Tanzania, it was concluded that for the majority of maternal deaths, the leading contributory factor, were from third phase delay due to both delay in treatment and inadequate skills of providers [34,35]. In general, women’s health programs would benefit from regular application of a social autopsy module as part of VA for adult female deaths, as has been used for maternal, neonatal, and child deaths in several settings [36,37]. In addition, more research is required to obtain insights into the quality of care through health facility assessments.

Fifth, our findings only partly support the national survey results and global estimates. The all-cause mortality levels were close to the results from the surveys, but the Magu HDSS results suggest that the decline has stagnated in recent years, which cannot be shown yet with survey data. The levels and trends in the main cause groups among women 15–49 years were close to the estimates based on global models, and in line with what has been observed in other HDSS reports [6–13]. For maternal mortality, the MMR for the most recent period (281) was in the same range as the UN estimates for Tanzania in 2020 (238, 80% uncertainty interval: 174,381). The Magu HDSS MMR was also similar to the Tanzania DHS national results for the late nineties, but declined earlier and then remained at 281 in 2015–2022, while the Tanzania DHS 2022 showed a dramatic decline to 104 per 100,000 live births (95% CI 59,149) for the period 2016–2022. Although Magu HDSS is not nationally representative, mortality and fertility rates tend to be the same as those of mainland Tanzania, which raises concerns about the results of the DHS 2022. The key difference between the Magu HDSS estimates and those from the DHS is the estimated proportion of women who died during pregnancy or postpartum, which is improbably low in the DHS estimates for 2015–2022 at 6.1%. For Magu HDSS, this figure is 13.5% and is supported by the narratives alongside the verbal autopsy signs and symptoms, with all but one mentioning pregnancy or delivery. Our sensitivity analysis, assuming that all those that did not have a VA were not maternal deaths, still did not bring the MMR down to the DHS levels.

Our study was limited by the absence of medical certification of the cause of death. VA is a crude instrument based on proxy respondents’ recall of signs and symptoms, which generally does not allow for detailed assessment of causes of death. This was further compounded by the use of multiple instrument versions over time. Our analyses were limited to broad cause groups, HIV/TB, and maternal deaths, thus reducing the effects of misclassification. Another limitation concerns the generalizability of our findings. VA coverage varies over time, and we assume that the cause of death, place of death, and seeking treatment patterns are the same for those without a VA, which may not be true. Although Magu HDSS has been shown to be typical for much of the Tanzanian mainland [18], it is not nationally representative. The proximity of the population to Mwanza City (on average, 1 hour’s travel for most of the population during the study period), with a regional and zonal referral hospital, may have affected some of the results. In addition, the annual numbers of adult female deaths by cause in Magu HDSS are small, with implications for a detailed assessment of trends. We addressed this issue by using multi-year time periods, smoothing of annual trends, imputations for known event omissions, and sensitivity analyses.

Although there have been substantial declines in mortality, between 1995 and 2022, for women age 15–49 driven by deaths due to communicable diseases, women now face a double burden with similar shares of communicable and non-communicable or injury-related deaths. Maternal mortality remains a leading cause at 12%, while HIV/TB continues to be the since largest cause of death, with a fraction of deaths similar to all non-communicable diseases combined. The Tanzania Ministry of Health recognizes the need for further action, and national health sector strategic plans include reducing maternal mortality as one of its major objectives [38,39] through expanded access to quality obstetric and newborn emergency care, nationwide maternal death surveillance and response (since 2018), and maintaining high institutional delivery rates [40]. A continued focus on maternal mortality is necessary, even with a high proportion of deliveries now occurring in health facilities, as part of a primary health care approach that addresses all major causes of death among women of reproductive age.

## Supplementary Material

Female_mortality_Appendix_REVISION_clean.docx

STROBE_checklist_Female Mortality_GHA.docx

## Data Availability

The data that support the findings of this study are available upon reasonable request from TAZAMA, National Institute of Medical Research, Mwanza, Tanzania (mwanza@nimr.or.tz).
