# Supplementary material for: Cause-specific mortality transition among women of reproductive age with special reference to maternal mortality: the Magu health and demographic surveillance system, Tanzania, 1995–2022
Source: Glob Health Action. 2026 Mar 18;19(1):2611693. doi: 10.1080/16549716.2025.2611693 (PMC13003866; doi:10.1080/16549716.2025.2611693)
Supplement: Female_mortality_Appendix_REVISION_clean.docx [file ZGHA_A_2611693_SM2535.docx]

# Cause-specific mortality transition among women of reproductive ages with special reference to maternal mortality: the Magu Health and Demographic Surveillance System, Tanzania, 1995-2022 (Supplementary Materials)

Milly Marston^1^, Sophia Kagoye^2^, Jacqueline Materu^2^, Charles Mangya^2^, Jim Todd^1^, Mark Urassa^2^, Ties Boerma^3^

1 Department of Population Health, London School of Hygiene & Tropical Medicine

2 National Institute for Medical Research, Mwanza, Tanzania

3 Institute for Global Public Health, University of Manitoba

Contents

[Cause of death categories 3](#_Toc171698251)

[Mortality Rates 4](#_Toc171698252)

[Verbal Autopsy Coverage 5](#_Toc171698253)

[Cause of death 7](#_Toc171698254)

[Proportion of deaths to women aged 15-49 8](#_Toc171698255)

[Fertility rates for women aged 15-49 9](#_Toc171698256)

[Place of death 11](#_Toc171698257)

[Verbal Autopsy Narratives 14](#_Toc171698258)

## Cause of death inputs and categories

Table S1: The InsilcoVA inputs mapped onto the Magu HDSS questionnaires using the Magu specific standard questionnaire (pre-WHO) and the WHO 2008 questionnaire. Blue indicates there was an equivalent question in the questionnaire that mapped to the InSilicoVA input and grey there was no equivalent.

|  |  | Pre-WHO | WHO |  |  | Pre-WHO | WHO |  |  | Pre-WHO | WHO |
| --- | --- | --- | --- | --- | --- | --- | --- | --- | --- | --- | --- |
| InSilivoVA input | i077o | 1 | 1 |  | i170o | 1 | 1 |  | i228o | 1 | 1 |
|  | i079o | 1 | 1 |  | i171o | 1 | 1 |  | i229o | 0 | 0 |
|  | i082o | 0 | 0 |  | i173a | 1 | 1 |  | i230o | 1 | 1 |
|  | i083o | 1 | 1 |  | i174o | 1 | 1 |  | i231o | 0 | 0 |
|  | i084o | 1 | 1 |  | i175o | 0 | 0 |  | i232a | 0 | 0 |
|  | i085o | 1 | 1 |  | i176a | 0 | 0 |  | i233o | 1 | 1 |
|  | i086o | 1 | 1 |  | i178a | 1 | 1 |  | i234a | 1 | 1 |
|  | i087o | 1 | 1 |  | i181o | 1 | 1 |  | i234b | 1 | 1 |
|  | i089o | 1 | 1 |  | i182a | 1 | 1 |  | i235a | 1 | 1 |
|  | i090o | 1 | 1 |  | i182b | 1 | 1 |  | i235b | 1 | 1 |
|  | i091o | 0 | 0 |  | i182c | 1 | 1 |  | i235c | 1 | 1 |
|  | i092o | 0 | 0 |  | i186o | 1 | 1 |  | i235d | 1 | 0 |
|  | i093o | 0 | 0 |  | i187o | 0 | 0 |  | i236o | 1 | 1 |
|  | i094o | 0 | 0 |  | i188o | 1 | 1 |  | i237o | 1 | 1 |
|  | i095o | 0 | 0 |  | i189o | 0 | 0 |  | i238o | 1 | 1 |
|  | i096o | 0 | 0 |  | i190o | 0 | 0 |  | i239o | 0 | 0 |
|  | i098o | 0 | 0 |  | i191o | 1 | 1 |  | i240o | 0 | 0 |
|  | i099o | 1 | 1 |  | i192o | 1 | 0 |  | i241o | 0 | 0 |
|  | i100o | 1 | 1 |  | i193o | 1 | 1 |  | i242o | 0 | 0 |
|  | i120a | 1 | 1 |  | i194o | 1 | 1 |  | i243o | 1 | 1 |
|  | i120b | 1 | 1 |  | i195o | 1 | 0 |  | i244o | 1 | 1 |
|  | i123o | 1 | 1 |  | i197a | 1 | 1 |  | i245o | 1 | 1 |
|  | i125o | 1 | 0 |  | i197b | 1 | 1 |  | i246o | 1 | 1 |
|  | i127o | 1 | 1 |  | i199a | 1 | 0 |  | i247o | 1 | 1 |
|  | i128o | 0 | 0 |  | i199b | 1 | 0 |  | i248a | 1 | 1 |
|  | i129o | 0 | 0 |  | i200o | 1 | 1 |  | i249o | 1 | 1 |
|  | i147o | 1 | 1 |  | i201a | 1 | 1 |  | i250a | 0 | 0 |
|  | i148a | 1 | 1 |  | i201b | 1 | 1 |  | i251o | 1 | 1 |
|  | i148b | 1 | 1 |  | i203a | 1 | 1 |  | i252o | 1 | 1 |
|  | i148c | 1 | 1 |  | i204o | 1 | 1 |  | i253o | 1 | 1 |
|  | i149o | 0 | 0 |  | i205a | 1 | 1 |  | i254o | 0 | 0 |
|  | i150a | 1 | 0 |  | i205b | 1 | 1 |  | i255o | 1 | 1 |
|  | i151a | 1 | 1 |  | i207o | 1 | 1 |  | i256o | 1 | 1 |
|  | i152o | 1 | 1 |  | i208o | 1 | 1 |  | i257o | 1 | 1 |
|  | i153o | 1 | 1 |  | i209a | 0 | 0 |  | i258o | 1 | 1 |
|  | i154a | 1 | 1 |  | i209b | 0 | 0 |  | i259o | 1 | 1 |
|  | i154b | 1 | 1 |  | i210o | 1 | 1 |  | i260a | 0 | 0 |
|  | i155o | 1 | 1 |  | i211a | 1 | 1 |  | i260b | 0 | 0 |
|  | i156o | 1 | 1 |  | i212o | 1 | 1 |  | i260c | 1 | 1 |
|  | i157o | 1 | 1 |  | i213o | 1 | 1 |  | i260d | 0 | 0 |
|  | i158o | 0 | 0 |  | i214o | 1 | 1 |  | i260e | 0 | 0 |
|  | i159o | 1 | 1 |  | i215o | 1 | 1 |  | i260f | 0 | 0 |
|  | i161a | 1 | 0 |  | i216a | 0 | 0 |  | i260g | 0 | 0 |
|  | i165a | 0 | 0 |  | i217o | 1 | 1 |  | i261o | 1 | 1 |
|  | i166o | 1 | 0 |  | i218o | 0 | 0 |  | i262a | 1 | 1 |
|  | i167a | 0 | 0 |  | i219o | 1 | 1 |  | i263a | 1 | 1 |
|  | i167b | 0 | 0 |  | i221a | 0 | 0 |  | i263b | 1 | 1 |
|  | i168o | 1 | 1 |  | i221b | 0 | 0 |  | i264o | 1 | 1 |
|  | i169a | 1 | 1 |  | i222o | 0 | 0 |  | i265o | 1 | 1 |
|  | i169b | 1 | 1 |  | i223o | 1 | 1 |  | i266a | 1 | 1 |
|  |  |  |  |  | i224o | 1 | 1 |  | i267o | 1 | 1 |
|  |  |  |  |  | i225o | 1 | 1 |  | i268o | 1 | 1 |
|  |  |  |  |  | i226o | 1 | 1 |  | i270o | 1 | 1 |
|  |  |  |  |  | i227o | 1 | 1 |  |  |  |  |

Table S2: Cause of death from InSilicoVA and broad cause of death groupings

| **InSilicoVA Cause of death** | **Broad cause of death** |
| --- | --- |
| Sepsis (non-obstetric) | Communicable |
| Acute resp infect incl pneumonia |  |
| HIV/AIDS related death |  |
| Diarrhoeal diseases |  |
| Malaria |  |
| Measles |  |
| Meningitis and encephalitis |  |
| Tetanus |  |
| Pulmonary tuberculosis |  |
| Pertussis |  |
| Haemorrhagic fever (non-dengue) |  |
| Dengue fever |  |
| Other and unspecified infect disease |  |
| Oral neoplasms | Non-Communicable |
| Digestive neoplasms |  |
| Respiratory neoplasms |  |
| Breast neoplasms |  |
| Reproductive neoplasms MF |  |
| Other and unspecified neoplasms |  |
| Severe anaemia |  |
| Severe malnutrition |  |
| Diabetes mellitus |  |
| Acute cardiac disease |  |
| Stroke |  |
| Sickle cell with crisis |  |
| Other and unspecified cardiac dis |  |
| Chronic obstructive pulmonary dis |  |
| Asthma |  |
| Acute abdomen |  |
| Liver cirrhosis |  |
| Renal failure |  |
| Epilepsy |  |
| Other and unspecified NCD |  |
| Ectopic pregnancy | Direct Obstetric Causes |
| Abortion-related death |  |
| Pregnancy-induced hypertension |  |
| Obstetric haemorrhage |  |
| Obstructed labour |  |
| Pregnancy-related sepsis |  |
| Anaemia of pregnancy |  |
| Ruptured uterus |  |
| Other and unspecified maternal CoD |  |
| Road traffic accident | External Cause of Death |
| Other transport accident |  |
| Accid fall |  |
| Accid drowning and submersion |  |
| Accid expos to smoke fire & flame |  |
| Contact with venomous plant/animal |  |
| Accid poisoning & noxious subs |  |
| Intentional self-harm |  |
| Assault |  |
| Exposure to force of nature |  |
| Other and unspecified external CoD |  |
| Indeterminate | Indeterminate |
| Algorithm could not process |  |

##

## Mortality Rates

Table S3: Annual deaths, person years and mortality rates for women age 15-49. Smoothed rate is based on a five year running average.

| **Year** | **Deaths** | **Person years per 1000** | **Mortality Rate per 1000** | **Smooth Rate per 1000** |
| --- | --- | --- | --- | --- |
| 1994* | 17 | 2138.5 | 8.0 |  |
| 1995 | 32 | 4179.1 | 7.7 |  |
| 1996 | 35 | 4319.4 | 8.1 | 7.78 |
| 1997 | 42 | 4442.5 | 9.5 | 8.04 |
| 1998 | 26 | 4520.9 | 5.8 | 8.81 |
| 1999 | 41 | 4444.6 | 9.2 | 9.02 |
| 2000 | 57 | 4948.4 | 11.5 | 8.85 |
| 2001 | 45 | 4929.4 | 9.1 | 9.33 |
| 2002 | 42 | 4864.9 | 8.6 | 8.66 |
| 2003 | 45 | 5525.1 | 8.1 | 7.90 |
| 2004 | 35 | 5954.9 | 5.9 | 6.80 |
| 2005 | 43 | 5589.7 | 7.7 | 6.76 |
| 2006 | 20 | 5466.9 | 3.7 | 6.11 |
| 2007 | 46 | 5466.2 | 8.4 | 6.03 |
| 2008 | 31 | 6324.4 | 4.9 | 5.52 |
| 2009 | 37 | 6757.2 | 5.5 | 5.54 |
| 2010 | 32 | 6205.7 | 5.2 | 4.57 |
| 2011 | 21 | 5616.7 | 3.7 | 4.72 |
| 2012 | 24 | 6708.1 | 3.6 | 4.40 |
| 2013 | 38 | 6705.3 | 5.7 | 3.79 |
| 2014 | 25 | 6452.3 | 3.9 | 3.52 |
| 2015 | 14 | 6709.3 | 2.1 | 3.34 |
| 2016 | 16 | 6702.9 | 2.4 | 3.05 |
| 2017 | 16 | 5978.9 | 2.7 | 3.17 |
| 2018 | 33 | 7799.0 | 4.2 | 3.36 |
| 2019 | 39 | 8758.0 | 4.5 | 3.53 |
| 2020 | 28 | 9221.3 | 3.0 | 3.49 |
| 2021 | 34 | 10000.0 | 3.2 |  |
| 2022 | 26 | 11000.0 | 2.5 |  |

*Partial year

## Verbal Autopsy Coverage

Table S4: VA coverage and known pregnancy status

| **Year** | **Number of deaths** | **Number with VA** | **VA coverage %** | **Pregnant or Postpartum in VA (n)** | **Not pregnant not postpartum in VA (n)** | **Pregnancy status unknown***  **(n)** | **Known Pregnancy Status %** |
| --- | --- | --- | --- | --- | --- | --- | --- |
| 1995 | 32 | 30 | 93.8 | 2 | 22 | 8 | 75.0 |
| 1996 | 35 | 30 | 85.7 | 5 | 18 | 12 | 65.7 |
| 1997 | 42 | 36 | 85.7 | 1 | 32 | 9 | 78.6 |
| 1998 | 26 | 23 | 88.5 | 3 | 19 | 4 | 84.6 |
| 1999 | 41 | 27 | 65.9 | 4 | 22 | 15 | 63.4 |
| 2000 | 57 | 36 | 63.2 | 8 | 27 | 22 | 61.4 |
| 2001 | 45 | 31 | 68.9 | 3 | 26 | 16 | 64.4 |
| 2002 | 42 | 30 | 71.4 | 4 | 26 | 12 | 71.4 |
| 2003 | 45 | 16 | 35.6 | 1 | 15 | 29 | 35.6 |
| 2004 | 35 | 13 | 37.1 | 1 | 13 | 21 | 40.0 |
| 2005 | 43 | 34 | 79.1 | 3 | 31 | 9 | 79.1 |
| 2006 | 20 | 11 | 55.0 | 1 | 10 | 9 | 55.0 |
| 2007 | 46 | 36 | 78.3 | 3 | 33 | 10 | 78.3 |
| 2008 | 31 | 26 | 83.9 | 4 | 22 | 5 | 83.9 |
| 2009 | 37 | 29 | 78.4 | 1 | 27 | 9 | 75.7 |
| 2010 | 32 | 23 | 71.9 | 4 | 19 | 9 | 71.9 |
| 2011 | 21 | 14 | 66.7 | 0 | 14 | 7 | 66.7 |
| 2012 | 24 | 21 | 87.5 | 4 | 17 | 3 | 87.5 |
| 2013 | 38 | 33 | 86.8 | 1 | 31 | 6 | 84.2 |
| 2014 | 25 | 20 | 80.0 | 0 | 20 | 5 | 80.0 |
| 2015 | 14 | 14 | 100.0 | 1 | 12 | 1 | 92.9 |
| 2016 | 16 | 14 | 87.5 | 1 | 13 | 2 | 87.5 |
| 2017 | 16 | 15 | 93.8 | 3 | 12 | 1 | 93.8 |
| 2018 | 33 | 28 | 84.8 | 5 | 22 | 6 | 81.8 |
| 2019 | 39 | 36 | 92.3 | 1 | 33 | 5 | 87.2 |
| 2020 | 28 | 23 | 82.1 | 4 | 19 | 5 | 82.1 |
| 2021 | 34 | 22 | 64.7 | 6 | 16 | 12 | 64.7 |
| 2022 | 26 | 25 | 96.2 | 3 | 22 | 1 | 96.2 |

*Pregnancy Status unknown can be didn’t have a VA or it was not specified in the VA

Table S5: VA coverage and known pregnancy status in three broad periods used for maternal mortality analysis

| **Year** | **Number of deaths** | **Number with VA** | **VA coverage %** | **Pregnancy Status** | | | **Known Pregnancy Status %** |
| --- | --- | --- | --- | --- | --- | --- | --- |
|  |  |  |  | **Pregnant or Postpartum (n)** | **Not pregnant not postpartum (n)** | **Pregnancy status unknown (n)** |  |
| 1995-2002 | 318 | 243 | 76.4 | 30 | 191 | 97 | 69.5 |
| 2005-2011 | 230 | 173 | 75.2 | 16 | 156 | 58 | 74.8 |
| 2015-2022 | 206 | 177 | 85.9 | 24 | 149 | 33 | 84.0 |

## Cause of death

Table S6: Cause specific mortality rates per 1000 person years for women aged 15-49 by calendar period

| Calendar Period | Deaths in HDSS  (n) | Cause specific mortality rate per 1000 person years | | | | |
| --- | --- | --- | --- | --- | --- | --- |
|  |  | HIV/TB | Communicable | Non-communicable disease | Maternal Causes | External Cause of Death |
| 1995-2001 | 278 | 4.46 | 1.34 | 1.66 | 1.00 | 0.28 |
| 2002-2008 | 262 | 2.09 | 0.99 | 2.48 | 0.50 | 0.63 |
| 2009-2015 | 191 | 1.79 | 0.59 | 1.23 | 0.17 | 0.42 |
| 2016-2022 | 192 | 1.09 | 0.34 | 1.12 | 0.40 | 0.28 |

Table S7: Cause specific mortality fractions for women aged 15-49 using aggregated probabilities from InSilicoVA

| Calendar Period | Deaths with VA (n) | Cause specific mortality fraction (%) | | | | |
| --- | --- | --- | --- | --- | --- | --- |
|  |  | HIV/TB | Communicable | Non-communicable disease | Maternal Causes | External Cause of Death |
| 1995-2001 | 213 | 51.0 | 15.3 | 19.0 | 11.5 | 3.2 |
| 2002-2008 | 166 | 31.2 | 14.8 | 37.2 | 7.5 | 9.4 |
| 2009-2015 | 154 | 42.6 | 14.0 | 29.3 | 4.0 | 10.1 |
| 2016-2022 | 163 | 33.7 | 10.5 | 34.8 | 12.4 | 8.6 |

Table S8: Cause specific mortality rates per 1000 person years for women aged 15-49 by calendar period

| **Cause of death** | **Calendar Period** | | | |
| --- | --- | --- | --- | --- |
|  | **1995-2001** | **2002-2008** | **2009-2015** | **2016-2022** |
| Acute resp infect incl pneumonia | 0.37 | 0.30 | 0.12 | 0.08 |
| HIV/AIDS/TB related death | 4.46 | 2.09 | 1.79 | 1.09 |
| Malaria | 0.03 | 0.03 | 0.00 | 0.00 |
| Meningitis and encephalitis | 0.34 | 0.22 | 0.24 | 0.08 |
| Other and unspecified infect dis | 0.60 | 0.44 | 0.22 | 0.17 |
| Neoplasms | 0.53 | 1.26 | 0.41 | 0.40 |
| Nutritional and Endocrine disorders | 0.11 | 0.03 | 0.01 | 0.06 |
| Diseases of circulatory system | 0.31 | 0.15 | 0.10 | 0.08 |
| Acute abdomen | 0.35 | 0.64 | 0.45 | 0.30 |
| Renal failure | 0.07 | 0.06 | 0.03 | 0.07 |
| Epilepsy | 0.15 | 0.19 | 0.09 | 0.07 |
| Maternal causes | 1.00 | 0.50 | 0.20 | 0.40 |
| External CoD | 0.28 | 0.63 | 0.42 | 0.28 |
| Other and unspecified NCD | 0.15 | 0.15 | 0.14 | 0.13 |

Table S9: Cause specific mortality fractions for women aged 15-49 using aggregated probabilities from InSilicoVA in more detailed categories

| **Cause of death** | **Calendar Period** | | | |
| --- | --- | --- | --- | --- |
|  | **1995-2001** | **2002-2008** | **2009-2015** | **2016-2022** |
| Acute respiratory infections incl pneumonia | 4.3 | 4.6 | 2.9 | 2.6 |
| HIV/AIDS/TB related death | 51.0 | 31.2 | 42.6 | 33.7 |
| Malaria | 0.3 | 0.4 | 0.0 | 0.0 |
| Meningitis and encephalitis | 3.9 | 3.2 | 5.7 | 2.6 |
| Other and unspecified infect dis | 6.8 | 6.6 | 5.3 | 5.3 |
| Neoplasms | 6.0 | 18.8 | 9.8 | 12.4 |
| Nutritional and Endocrine disorders | 1.3 | 0.4 | 0.1 | 1.8 |
| Diseases of circulatory system | 3.6 | 2.3 | 2.3 | 2.6 |
| Acute abdomen | 4.0 | 9.6 | 10.7 | 9.3 |
| Renal failure | 0.8 | 0.9 | 0.6 | 2.2 |
| Epilepsy | 1.7 | 2.9 | 2.3 | 2.3 |
| Maternal causes | 11.5 | 7.5 | 4.0 | 12.4 |
| Other and unspecified NCD | 1.7 | 2.2 | 3.4 | 4.1 |
| External CoD | 3.2 | 9.4 | 10.1 | 8.6 |
| Total | 100.0 | 100.0 | 100.0 | 100.0 |

## Proportion of deaths to women aged 15-49

Table S10: Percentage of female deaths that are pregnancy related by calendar periods and DHS comparison. NB this is equivalent to maternal related deaths apart from age group 20-34 in 2005-2011 where one death was from external causes.

|  | **1995-2002** | | |  | **2005-2011** | | |  | **2015-2022** | | |  | **DHS 2015-2022** | | |
| --- | --- | --- | --- | --- | --- | --- | --- | --- | --- | --- | --- | --- | --- | --- | --- |
| **Age Group** | **Total Known** | **PregPP** | **Percentage of female deaths that are maternal** |  | **Total Known** | **PregPP** | **Percentage of female deaths that are maternal** |  | **Total Known** | **PregPP** | **Percentage of female deaths that are maternal** |  | **Total Known** | **PregPP** | **Percentage of female deaths that are maternal** |
| **15-19** | 17 | 6 | 35.3 |  | 17 | 2 | 11.8 |  | 16 | 2 | 12.5 |  | 25 | 2 | 8.0 |
| **20-24** | 35 | 7 | 20.0 |  | 13 | 2 | 15.4 |  | 15 | 3 | 20.0 |  | 46 | 7 | 15.2 |
| **25-29** | 44 | 2 | 4.5 |  | 34 | 6 | 17.6 |  | 20 | 6 | 30.0 |  | 69 | 2 | 2.9 |
| **30-34** | 43 | 5 | 11.6 |  | 31 | 4* | 12.9 |  | 36 | 5 | 13.9 |  | 97 | 6 | 6.2 |
| **35-39** | 33 | 7 | 21.2 |  | 27 | 0 | 0.0 |  | 30 | 5 | 16.7 |  | 99 | 9 | 9.1 |
| **40-44** | 27 | 2 | 7.4 |  | 28 | 2 | 7.1 |  | 33 | 2 | 6.1 |  | 86 | 3 | 3.5 |
| **45-49** | 23 | 1 | 4.3 |  | 22 | 0 | 0.0 |  | 23 | 1 | 4.3 |  | 63 | 1 | 1.6 |
| **Total** | **222** | **30** | **13.5** |  | **172** | **16*** | **9.3** |  | **173** | **24** | **13.9** |  | **492** | **30** | **6.1** |

***This includes one external cause of death**

## Fertility rates for women aged 15-49

Fertility was under enumerated in the periods 2012-2016 and 2021-2022 (Figure A4) in order to calculate the MMR total number of births in the period of choice are required. In order to impute the years with under enumeration we fitted a line using a least squares fit through the annual smoothed rates of 2010, 2017-2020. The annual births and imputed annual births for these specific years, were then aggregated for the three broad periods used in the analysis ready to calculate the MMR.

Figure S1: General fertility rate women aged 15-49, annual rates, annual rates with imputed years 2012-2016 and 2021-2022, smoothed rate of a 5 year running average compared to national estimates from the Tanzanian DHS

Table S11: Total number of births to women aged 15-49 recorded in calendar periods and number of imputed births (where 2012-2016 and 2021-2022 GFR is imputed)

| **Calendar Period** | **Births in period** | **Imputed births in period** |
| --- | --- | --- |
| 1995-2002 | 7379 | 7379 |
| 2005-2011 | 7092 | 7092 |
| 2015-2022 | 8627 | 9771 |

#### **Maternal Mortality Ratio Sensitivity analysis**

Figure S2: Maternal mortality ratio estimates by different general fertility rate scenarios

## Verbal Autopsy Narratives

Table S12: Selection of VA variables, Cause of death assigned and Narratives of women who had maternal related deaths 2015-2022

| **Five year age group** | **Pregnancy Status at death** | **Where did they deliver** | **Delivery method** | **Place of death** | **Notes from questionnaire section of VA** | **Narrative- Summary** | **Summary of Physcian review** | **Final Physcian CoD** |
| --- | --- | --- | --- | --- | --- | --- | --- | --- |
| 40-44 | Died <6 weeks after early pregnancy ending | . | . | Hospital | Ill for 12 months, HIV positive, Had fever for 12 months on and off, severe cough for 10 months, chest pain, diarrhoea for two months, abdominal pain for four months, headache, weight loss, recent abortion. Received oral rehydration. Had ever tested for HIV. | Had HIV, was pregnant. Aborted on purpose, health deteriorated, dies in in hospital five days after admission. | Physician 1: Induced abortion  Physician 2: Induced abortion | Induced abortion |
| 15-19 | Died within 6 weeks after normal length | Hospital | Normal vaginal | Hospital | Abdominal pain for 14 days, bled post labour, received IV, blood transfusion. | Nine Months pregnant, delivered child safely. She had severe abdominal pain and heavy vaginal bleeding two days after delivery. Traditional medicines used, but did not work so took her to the hospital, referred to another hospital after five days. Given a blood transfusion, but she got worse and died. | Physician 1: Postpartum haemorrhage Physician 2: Postpartum haemorrhage | Postpartum haemorrhage |
| 25-29 | Died within 6 weeks after normal length | Health centre | Normal vaginal | Home | Ill for seven days, anaemia, bled post labour | Nine Months pregnant with second pregnancy. Delivered safely and left hospital. At home she had heavy vaginal bleeding and returned to hospital. Given oral medication to treat anaemia and returned home. Her condition worsened and she died one week later. | Physician 1: Postpartum haemorrhage Physician 2: Postpartum haemorrhage | Postpartum haemorrhage |
| 30-34 | Pregnant at time of death | . | . | Hospital | More than four previous pregnancies, Sudden coma | At two months pregnant, she had sudden abdominal pain, vaginal bleeding and lost consciousness. Taken to clinic and referred to hospital. Tested and found that the pregnancy was implanted outside her womb. | Physician 1: Ectopic Pregnancy Physician 2: Ectopic Pregnancy | Ectopic pregnancy |
| 45-49 | Died within 6 weeks after normal length | Home | Normal vaginal | Health Centre | More than four previous pregnancies, Anaemia, bled post labour, placenta retained. | Nine months pregnant, she delivered at home at 6:00pm she bled heavily and the placenta did not come out. At 8:00am the next day she was taken to hospital. The hospital was far away and just after arriving there, she died. | Physician 1: Postpartum haemorrhage secondary to Retained placenta  Physician 2: Postpartum haemorrhage secondary to Retained placenta | Postpartum Haemorrhage |
| 15-19 | Pregnant at time of death | . | . | Hospital | First pregnancy | Six months pregnant at time of death. During pregnancy she had a history of fever and vomiting. She was admitted to the clinic for two weeks, after not improving she was referred to the hospital and diagnosed with anaemia. | Physician 1: Anaemia in pregnancy Physician 2: Malaria, with severe anemia as consequence | Anaemia in pregnancy |

Table S11: continued

| **Five year age group** | **Pregnancy Status at death** | **Where did they deliver** | **Delivery method** | **Place of death** | **Notes from questionnaire section of VA** | **Narrative- Summary** | **Summary of Physcian review** | **Final Physcian CoD** |
| --- | --- | --- | --- | --- | --- | --- | --- | --- |
| 20-24 | Died within 6 weeks after normal length | Health centre | Normal vaginal | Hospital | First pregnancy, Labour over 24 hours, dies within 24 hours of delivery, placenta retained. | Pregnant with twins. At the due date she went to the health centre to deliver. She went into labour at 1:00pm, the first twin was delivered at 4:00am the next morning and the second at 2:00pm. After delivering the second twin the deceased was very tired and two hours later passed away. | Physician 1: Prolonged/ obstructed labour and retained placenta  Physician 2: Prolonged/ obstructed labour and retained placenta | Prolonged/ obstructed labour and retained placenta |
| 25-29 | Died within 6 weeks after normal length | Hospital | Normal vaginal | Hospital | Ill for three days, anaemia, bleeding post delivery. | The deceased was travelling for business. The news received was that she died three days after giving birth in hospital. After giving birth she bled heavily which is what caused her death. | Physician 1: Postpartum haemorrhage  Physician 2: Postpartum haemorrhage | Postpartum haemorrhage |
| 30-34 | Died within 6 weeks after normal length | Home | Normal vaginal | Other | More than four previous pregnancies, Died within 24 hours of delivery, placenta retained | Nine months pregnant she decided to deliver at home. She delivered the baby but the placenta was retained. After waiting awhile for the placenta to come out, without success, she was taken to hospital to get further assistance. She died on the way to the hospital. | Physician 1: Postpartum haemorrhage secondary to Retained placenta  Physician 2: Postpartum haemorrhage secondary to Retained placenta | Postpartum Haemorrhage |
| 35-39 | Pregnant at time of death | . | . | Health Centre | ill for five months, fever on and off for two months, jaundice, headache, sudden coma | She had lower abdominal pain from the beginning of the pregnancy. It was ten years since she last gave birth. This was her fourth child and she attended the clinic many times for treatment. At six months pregnant she was referred to the health centre, there she was not able to speak anymore. She was given intravenous fluids and at 11:00pm she died. | Physician 1: Unknown  Physician 2: Unknown possible Eclampsia? | Unknown |
| 40-44 | Pregnant at time of death | . | . | Hospital | HIV positive, fever on and off for 12 months, cough, jaundiced for three months, diarrhoea for four months, vomiting for a week. Abdominal pain for 24 hours, headache. | The deceased had HIV | Physician 1: HIV/AIDS  Physician 2: HIV/AIDS aggravated by pregnancy | HIV |
| 20-24 | Pregnant at time of death | . | . | Hospital | Anaemia, excessive vaginal bleeding, recent abortion. Received IV | The deceased had abdominal pain, bleeding from the vaginal and the mouth. At the health centre she was tested positive for pregnancy and they suspected an induced abortion. She bled heavily and died at the health centre | Physician1: Induced Abortion  Physician2: Induced Abortion | Induced abortion |

Table S11: continued

| **Five year age group** | **Pregnancy Status at death** | **Where did they deliver** | **Delivery method** | **Place of death** | **Notes from questionnaire section of VA** | **Narrative- Summary** | **Summary of Physcian review** | **Final Physcian CoD** |
| --- | --- | --- | --- | --- | --- | --- | --- | --- |
| 20-24 | Died within 6 weeks after normal length | Hospital | Normal vaginal | Home | ill for 12 months, HIV positive, Had fever for 3 months on and off, severe cough for 12 months, breathlessness for three days, chest pain, jaundiced, diarrhoea for one months, vomiting for seven days, abdominal pain for three months, headache, swollen ankles, anaemia, received oral rehydration. On ART for about 6 months. | She had abdominal pain, vomiting, fever, cough, chest pain, swollen lower limbs and diarrhoea. She was treated at the health centre and multiple hospitals. She was diagnosed with HIV and was on medication. She died at home. | Physician 1: HIV  Physician 2: HIV with possibly induced hypertension. | HIV |
| 25-29 | Died within 6 weeks after normal length | Hospital | Normal vaginal | Hospital | Breathlessness for 2 days lying flat and on exertion, chest pain. Received treatment. | The baby was delivered safely. 11 days after delivery, in the night, she had a tightness in her chest and difficulty breathing. The next morning she was taken at 9:00am to the health centre where she was admitted. At 1:00pm the same day she died. | Physician 1: Pulmonary embolism  Physician 2: Pulmonary embolism | Pulmonary embolism |
| 25-29 | Pregnant at time of death | . | . | Hospital | Ill for three months, vomiting for seven days, anaemia, received IV, blood transfusion. | The deceased had boils in the armpits and abdominal pain | Physician 1: Unknown  Physician 2: Unknown | unknown |
| 25-29 | Died within 6 weeks after normal length | Hospital | Normal vaginal | Hospital | Ill for 14 days, fever on and off for 14 days, headache, coma, convulsions, weight loss, foul smelling vaginal discharge. Note abortion at 7 months is also recorded. | She had high blood pressure and a history of convulsions during pregnancy. The high blood pressure started while pregnant, she was taken to the health centre and then referred to the hospital. She was admitted for two weeks and then died. | Physician 1: Eclampsia  Physician 2: Eclampsia | Eclampsia |
| 30-34 | Died within 6 weeks after normal length | Hospital | Normal vaginal | Hospital | HIV Positive, Jaundiced for seven months, had anaemia, paralysed legs. On ART, received iv, blood transfusion. | She was four months pregnant when both her legs were paralysed. After delivery she died two weeks after this. | Physician 1: Pulmonary embolism (secondary to deep vein thrombosis)  Physician 2: Pulmonary embolism (possibly secondary to sepsis) | Pulmonary embolism |
| 35-39 | Died within 6 weeks after normal length | Hospital | Normal vaginal | Hospital | More than four previous pregnancies, Died in labour, excessive vaginal bleeding, placenta retained | She died while giving birth, she bled heavily. | Physician 1: Postpartum haemorrhage Physician 2: Postpartum haemorrhage | Postpartum haemorrhage |

Table S11: Continued

| **Five year age group** | **Pregnancy Status at death** | **Where did they deliver** | **Delivery method** | **Place of death** | **Notes from questionnaire section of VA** | **Narrative- Summary** | **Summary of Physcian review** | **Final Physcian CoD** |
| --- | --- | --- | --- | --- | --- | --- | --- | --- |
| 30-34 | Died within 6 weeks after normal length | Hospital | Normal vaginal | Hospital | Coma, Anaemia, labour over 24 hours, Bled post labour, received IV, blood transfusion. | The deceased was healthy. She went to the hospital to give birth when she was due. During delivery she bled heavily but she recovered and returned home. Two days later she changed and was tired and had no energy. She returned to hospital unconscious and was treated. A week later she died at the hospital. | Physician 1: Postpartum haemorrhage (following severe anaemia)  Physician 2: Postpartum haemorrhage | Postpartum haemorrhage |
| 30-34 | Died within 6 weeks after normal length | Health centre | Normal vaginal | Home | Received treatment | She had high blood pressure during pregnancy. She was taken to hospital for delivery where she did well and went home. The following Sunday her condition changed, she went back to hospital and was treated for high blood pressure and given other medication. She did well and returned home. The next day she was found dead at home. | Physician 1: Pre-eclampsia Physician 2: Pre-eclampsia | Pre-eclampsia |
| 40-44 | Died within 6 weeks after normal length | Health centre | Normal vaginal | Home |  | She was healthy. She finished eating went to sleep and was found dead in the morning with no complaints about being sick. | Physician 1: Unknown Physician 2: Unknown | unknown |

## Place of death

Table S13: Numbers for place of death analysis, women aged 15-49 years old, note that don’t know for all women is either no VA or the VA is don’t know. For known pregnant/PP women or not pregnant, don’t know is don’t know on the VA.

|  |  | **Place of Death** | | | | | | |  |
| --- | --- | --- | --- | --- | --- | --- | --- | --- | --- |
|  | **Period** | **Hospital** | **Health Centre** | **On route to hospital** | **Home** | **Traditional Healer** | **Other** | **Don't know** | **Total** |
| ***All women 15-49*** | |  |  |  |  |  |  |  |  |
|  | **2009-2015** | 55 | 1 | 3 | 73 | 11 | 7 | 42 | 192 |
|  | **2016-2022** | 71 | 1 | 3 | 73 | 1 | 8 | 35 | 192 |
|  |  |  |  |  |  |  |  |  |  |
|  | **Total** | 126 | 2 | 6 | 146 | 12 | 15 | 77 | 384 |
|  |  |  |  |  |  |  |  |  |  |
| ***Known not pregnant/PP women*** | | |  |  |  |  |  |  |  |
|  | **2009-2015** | 46 | 1 | 2 | 72 | 10 | 7 | 0 | 138 |
|  | **2016-2022** | 55 | 0 | 2 | 68 | 1 | 8 | 0 | 134 |
|  |  |  |  |  |  |  |  |  |  |
|  | **Total** | 101 | 1 | 4 | 140 | 11 | 15 | 0 | 272 |
|  |  |  |  |  |  |  |  |  |  |
| ***Known pregnant/PP women*** | | |  |  |  |  |  |  |  |
|  | **2009-2015** | 8 | 0 | 1 | 1 | 1 | 0 | 0 | 11 |
|  | **2016-2022** | 16 | 1 | 1 | 5 | 0 | 0 | 0 | 23 |
|  |  |  |  |  |  |  |  |  |  |
|  | **Total** | 24 | 1 | 2 | 6 | 1 | 0 | 0 | 34 |

Figure S3: Percentage of women who sought care for the illness that led up to the death by pregnancy status for years 2015-2022. Formal care includes health centre or hospital, informal care includes traditional healers, friends and pharmacies. This excludes deaths from external causes

Figure S4: Percentage of women who sought care for the illness that led up to the death by broad cause for years 2015-2022. Formal care includes health centre or hospital, informal care includes traditional healers, friends and pharmacies. This excludes deaths from external causes and those indeterminate (Communicable n=77, Non Communicable n=61, Direct obstetric causes n=21)

Figure S5: Percentage of women who sought care for the illness that led up to the death by place of death for years 2015-2022. Formal care includes health centre or hospital, informal care includes traditional healers and pharmacies. This excludes deaths from external causes and those indeterminate. (Hospital, n=70, Home n=75)
